# Bovine Placentome-Derived Extracellular Matrix: A Sustainable 3D Scaffold for Cultivated Meat

**DOI:** 10.3390/bioengineering11080854

**Published:** 2024-08-21

**Authors:** Cemile Bektas, Kathleen Lee, Anisha Jackson, Mohit Bhatia, Yong Mao

**Affiliations:** 1 Laboratory for Biomaterials Research, Department of Chemistry and Chemical Biology, Rutgers University, 145 Bevier Road, Piscataway, NJ 08854, USA; cemilekilic87@gmail.com (C.B.); kwl49@scarletmail.rutgers.edu (K.L.); atj43@scarletmail.rutgers.edu (A.J.); 2 Atelier Meats, 666 Burrard Street, Suite 500, Vancouver, BC V6C 3P6, Canada; mohitbhatia@hotmail.com

**Keywords:** tissue engineering, extracellular matrix, collagen, scaffolds, dehydrothermal crosslinking (DHT), cultivated meat

## Abstract

Cultivated meat, an advancement in cellular agriculture, holds promise in addressing environmental, ethical, and health challenges associated with traditional meat production. Utilizing tissue engineering principles, cultivated meat production employs biomaterials and technologies to create cell-based structures by introducing cells into a biocompatible scaffold, mimicking tissue organization. Among the cell sources used for producing muscle-like tissue for cultivated meats, primary adult stem cells like muscle satellite cells exhibit robust capabilities for proliferation and differentiation into myocytes, presenting a promising avenue for cultivated meat production. Evolutionarily optimized for growth in a 3D microenvironment, these cells benefit from the biochemical and biophysical cues provided by the extracellular matrix (ECM), regulating cell organization, interactions, and behavior. While plant protein-based scaffolds have been explored for their utilization for cultivated meat, they lack the biological cues for animal cells unless functionalized. Conversely, a decellularized bovine placental tissue ECM, processed from discarded birth tissue, achieves the biological functionalities of animal tissue ECM without harming animals. In this study, collagen and total ECM were prepared from decellularized bovine placental tissues. The collagen content was determined to be approximately 70% and 40% in isolated collagen and ECM, respectively. The resulting porous scaffolds, crosslinked through a dehydrothermal (DHT) crosslinking method without chemical crosslinking agents, supported the growth of bovine myoblasts. ECM scaffolds exhibited superior compatibility and stability compared to collagen scaffolds. In an attempt to make cultivate meat constructs, bovine myoblasts were cultured in steak-shaped ECM scaffolds for about 50 days. The resulting construct not only resembled muscle tissues but also displayed high cellularity with indications of myogenic differentiation. Furthermore, the meat constructs were cookable and able to sustain the grilling/frying. Our study is the first to utilize a unique bovine placentome-derived ECM scaffold to create a muscle tissue-like meat construct, demonstrating a promising and sustainable option for cultivated meat production.

## 1. Introduction

The current food system significantly impacts the environment, particularly with livestock production dominating global land use and contributing substantially to greenhouse gas emissions and climate change [1,2,3]. Beyond ethical considerations for animal welfare in industrial animal agriculture, there are noteworthy public health concerns, including antibiotic resistance and the potential for zoonotic disease risks associated with this practice [4,5,6]. Moreover, traditional animal agriculture faces challenges in meeting the growing demand for meat and protein as the global population expands [7,8]. Consequently, the last decade has witnessed accelerated innovation in cultivated meat and cellular agriculture, presenting a promising avenue to deliver authentic meat products while addressing environmental, ethical, and technical issues [9,10]. Cultivated meat, also known as cell-based or lab-grown meat, represents a revolutionary advancement in the field of food technology [11,12]. With industrial-scale production, cultivated meat is predicted to reduce water usage by 82–96%, land usage by 99%, and greenhouse gas emissions by up to 96% compared to conventional methods [13]. 

Cultivated meat production utilizes technologies and biomaterials originally designed for tissue engineering, a discipline centered on cells, signals, and scaffolds. In this process, cells are introduced into a biocompatible tissue scaffold for structural support, while essential nutrients guide their growth and function [12]. Through careful selection of agriculturally relevant cell sources, providing external signals, and utilization of supportive tissue scaffolds, the tissue engineering approach can be effectively applied to produce cultivated meat products. 

The primary focus in cultivated meat production lies in cell sourcing. To ensure successful production, the initial cell type must exhibit sufficient proliferative capabilities and subsequently differentiate into the mature cell types that constitute meat. Stem cells, particularly primary adult stem cells, emerge as the most promising candidates to achieve this objective [11]. Muscle satellite cells, employed in this study, positioned beneath the muscle fiber’s basement membrane, are stem cells with the ability to differentiate into myocytes. Subsequently, these myocytes form multinucleated myotubes, ultimately contributing to the creation of myofibers. Muscle satellite cells represent one of the most abundant adult stem cell populations within tissues, with well-established procedures for their isolation from livestock and in vitro maintenance [11,14]. Bovine muscle satellite cells have been extensively utilized in cultivated meat studies, as evidenced in the literature [15,16,17,18,19].

Selecting an optimal scaffolding material and acquiring suitable constructs are integral to the second stage of cultivated meat production. The literature highlights the prominent use of animal-derived [20,21], plant-derived [22,23,24,25], and synthetic polymers [26,27] as key biomaterials in this context. While animal-derived biomaterials, abundant in extracellular matrix (ECM) and supportive of cell growth, exhibit properties such as full absorption by the human body and structural similarity to conventional meat, their environmental and ethical considerations pose challenges to their sustainable acceptance [28]. 

To take advantage of the animal-derived ECM while avoiding harm to animals and ethical concerns, birth tissues, naturally expelled after birth, possess the desired properties. This study introduces a novel scaffolding material addressing environmental and ethical concerns: collagen and extracellular matrix (ECM) derived from bovine placentomes. Bovine placentomes serve as functional units of the placenta. Comprising a maternal cotyledon and a fetal caruncle, these structures play a vital role in facilitating the exchange of nutrients and gases between the maternal and fetal circulations throughout pregnancy [29]. Given the natural postpartum expulsion of the placenta, commonly known as ‘afterbirth’, harnessing ECM-rich bovine placentomes emerges as a viable and non-harmful strategy for advancing cultivated meat production (patent pending). Bovine placentomes can be ethically sourced from abattoirs and dairy farms thus minimizing waste and making efficient use of available materials.

The production of 3D scaffolds is a crucial step in cultivated meat production, ensuring efficient oxygen transport, waste removal, control of tissue geometry, cell distribution, and contributing to the final product’s structure [15,16]. Beyond being a structural support, 3D scaffolds offer biochemical and physical cues that mimic the microenvironment of natural tissues, potentially influencing the biology and behavior of cultivated cells, leading to a tissue structure more reminiscent of in vivo conditions and improved organoleptic properties. Additionally, scaffolds play a pivotal role in enhancing the scalability and cost-effectiveness of cultivated meat by contributing to the overall mass of the meat product [28,30]. An optimal scaffold for cultivated meat production should possess biocompatibility, high porosity, sufficient mechanical strength, and provide guidance for cell adhesion, proliferation, and differentiation [31,32]. Among diverse scaffolding strategies, such as microcarriers, fibers, hydrogels, and scaffold-free approaches, the porous scaffolds employed in this study exhibit cost-effective, straightforward, and scalable production, providing a robust framework for efficient cell proliferation. The tunable pore sizes allow for cell attachment, differentiation, and efficient transport of oxygen, nutrients, and waste [28,33]. The production of these porous scaffolds in this study involved the use of the freeze-drying technique, and the steak-like shape was successfully reproduced through the utilization of a 3D-printed mold.

Additional crucial considerations involve the biodegradability of materials and limitations on non-edible or toxic components, including solvents and crosslinking agents. Dehydrothermal (DHT) crosslinking is a physical crosslinking technique that involves the elimination of bound water from collagen molecules. This process induces condensation reactions between carboxyl and amino groups on adjacent amino acid side chains [34]. Notably, DHT offers an advantage over alternative crosslinking methods by avoiding the generation of potentially cytotoxic reaction products associated with the use of additional chemical reagents [35,36].

Our comparison study here demonstrated enhanced compatibility and stability of ECM scaffolds in contrast to collagen scaffolds. In the pursuit of cultivating meat constructs, bovine myoblasts were cultured within ECM scaffolds shaped like steaks for approximately 50 days. The resultant construct not only exhibited a resemblance to natural muscle tissues but also showcased elevated cellularity, signifying myogenic differentiation. Additionally, these meat constructs proved cookable and capable of withstanding grilling or frying. Our study represents the first use of a unique bioactive bovine placentome tissue ECM scaffold to create a meat construct resembling natural muscle tissue, offering a promising and sustainable approach to cultivated meat production.

## 2. Materials and Methods

### 2.1. Preparation of Bovine Placentome Tissue for Extraction

Following the retrieval of bovine placental tissues from the New Jersey Agricultural Experiment Station at Rutgers University in New Brunswick, NJ, USA, individual placentomes were isolated and stored in 50 mL conical tubes at −80 °C until further use. Thawed bovine placentomes underwent thorough washing with distilled water (diH_2_O) 3–4 times to remove blood and debris. The cleaned tissues were then immersed in 70% ethanol (1 g wet weight tissue to 4 mL solution ratio) for 30 min with agitation. After ethanol treatment, the tissues were rinsed twice with sterile diH_2_O, each for 15 min while mixing.

### 2.2. Extraction of Collagen from Bovine Placental Tissues

Cleaned tissues were frozen at −80 °C and lyophilized. To extract collagen, the lyophilized tissue was ground and mixed with distilled water at room temperature for 15 min, allowing the mixture to settle and the supernatant to be removed, and the process was repeated twice. The tissues were then soaked in 2 M NaCl solution overnight at 4 °C and washed three times with dH_2_O for 15 min each, with the final wash lasting 3 h at 4 °C for osmotic shock.

After removing the supernatant, tissues underwent 0.5 N and 2 N HCl treatment with mixing and incubation at room temperature for 75 min. The solution was neutralized with 2 N NaOH and diluted 10 times with dH_2_O, adjusting the pH to 1.5–2.3. A pepsin solution (10 mM HCl, 58 mg/mL) was added to reach a final concentration of 0.5 mg/mL, then mixed overnight at 4 °C. The pepsin concentration was increased to 2 mg/mL and mixed overnight at room temperature, keeping the pH between 1.5 and 2.3.

The digested solution, containing extracted atelopeptide collagen, was sequentially filtered using a vacuum system through a wire mesh and filters of 1 mm, 25 µm, 5–10 µm, and 5 µm. The filtrate was immersed in 0.75–0.2 M NaCl with 4 M NaCl at 4 °C overnight to precipitate collagen. The collagen precipitate was centrifuged at 3750 rpm (3200× *g*). The supernatant was discarded, and the collagen precipitates were placed in dialysis bags (SnakeSkin, 10K MWCO, ThermoFisher Scientific, Bridgewater, NJ, USA) and dialyzed against 10 mM HCl at 4 °C for 3–4 days. Once complete, the collagen solution was removed, frozen at −80 °C, and lyophilized until dry. 

### 2.3. Extraction of Extracellular Matrix (ECM) from Bovine Placental Tissues

After the initial tissue preparation, tissues were immersed in 1% SDS solution and mixed for 20 h at room temperature. SDS was removed by washing the tissues three times with distilled water for 30 min each while mixing. The tissues were then cut into small pieces (~1 cm × 1 cm × 1 cm) and incubated in 1% SDS for another 20 h at room temperature while mixing to remove cellular components. Afterward, they were washed three times with distilled water for 30 min each while mixing, followed by an overnight soak in distilled water at 4 °C.

The next day, tissues were washed twice for 30 min each with distilled water at room temperature on a shaker. The tissues were then frozen in a lyophilizing container at −80 °C, lyophilized until dry, and stored at 4 °C until use. 

To extract ECM, the lyophilized tissues were ground and digested in 0.5 M acetic acid with 2 mg/mL pepsin overnight at room temperature while mixing. Any small undigested tissues such as blood vessels or umbilical cords were removed using sterilized forceps. The solution was neutralized to pH 7 with 2.5 M NaOH.

**Figure 1:**
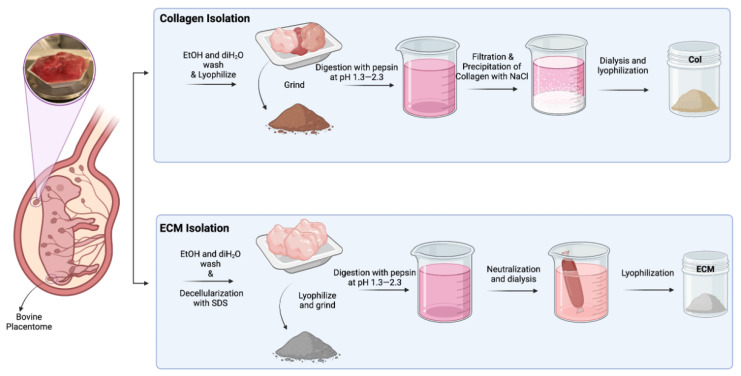
Schematic illustration of isolation of collagen and ECM from bovine placentomes.

The solution was transferred to SnakeSkin 10K MWCO dialysis tubing and dialyzed in 2 L of sterile distilled water at 4 °C for 3 days while stirring, changing the water twice daily. After dialysis, the solution was frozen at −20 °C and lyophilized (Figure 1).

### 2.4. Characterization of Collagen & ECM 

To quantify collagen content in isolated collagen and ECM, a hydroxyproline assay was conducted according to established protocols [37]. Briefly, 10 mg/sample of each material was digested in 500 µL Papain solution overnight at 60 °C. The Papain solution comprised 3.875 U/mL papain, 5 mM EDTA tetrasodium hydrate, 5 mM N-acetyl-L-cysteine, and 50 mM potassium phosphate buffer at pH 7. After digestion, samples were diluted 1:100 with water, processed with 100 µL of 4 N NaOH, and autoclaved. After the samples cooled to RT, 100 µL of 4 N HCl was added to neutralize the pH. Standard solutions (0, 2, 4, 6, 8, and 10 ug/tube) were prepared using a 1 mg/mL hydroxyproline stock solution (in diH_2_O), and 200 µL diH_2_O was added to each tube to ensure the same dilution with samples. A total of 625 µL Chloramine-T solution was added to each sample and incubated at RT for 20 min. Chloramine-T solution consisted of 0.05 M Chloramine-T in 74% *v*/*v* H_2_O 26% *v*/*v* 2-propanol, 0.629 M NaOH, 0.140 M citric acid (monohydrate), 0.453 M sodium acetate (anhydrous), and 0.112 M acetic acid. After 20 min, 625 µL Ehrlich’s solution was added to each sample and vortexed. Ehrlich’s solution consisted of 1 M DMAB in 30% *v*/*v* HCl, and 70% *v*/*v* 2-propanol incubated in a water bath at 65 °C for 20 min prior to use. Samples were incubated at 65 °C for 20 min and immediately quenched by immersing tubes in ice. Standards and samples were placed in triplicate in a 96-well clear, flat-bottomed plate (100 µL/well). Both standards and samples were analyzed spectrophotometrically at 560 nm. The absorbance was read in a plate reader (Spark^®^, TECAN, Männedorf, Switzerland) at an absorbance wavelength of 560 nm.

### 2.5. Production of Collagen and ECM Scaffolds

A total of 14 mg/mL BP-Collagen and ECM were prepared in 10 mM HCl separately, followed by overnight mixing at 4 °C. Subsequently, 750 µL/well of fully dissolved BP-Collagen or ECM was added to a 24-well plate coated with polydimethylsiloxane (PDMS) (Slygard 184, Dow Corning, Midland, MI, USA), frozen at −20 °C, and lyophilized. For thermal crosslinking, the scaffolds (diameter = 13 mm, height = 3 mm) were crosslinked in an Isotemp Vacuum Oven (Model 285A, Fisher Scientific, Pittsburgh, PA, USA) at 150 °C with a vacuum of −20 Hg for 24 h. This dehydrothermal crosslinking condition was optimized by previous studies [36,38]. For transglutaminase crosslinking, lyophilized and UV-sterilized scaffolds were incubated in 1 mL of 2.5% transglutaminase solution in PBS (Sigma-Aldrich, St. Louis, MO, USA) for 30 min. For comparison, thermal crosslinked scaffolds were incubated in 1 mL PBS. After 30 min, scaffolds were washed with 1 mL PBS and patted dry on UV-sterilized Kim Wipes.

### 2.6. Scanning Electron Microscopy

The scaffold sections were attached to aluminum sample stabs using double-sided conductive adhesive tape. These sample stabs were then positioned on a charge-reducing sample holder and loaded into the Scanning Electron Microscope (SEM) (Phenom ProX, Phenom World, 15 kV, Thermo Fisher Scientific, Somerset, NJ, USA). Images were captured at three different locations, using both low (500×) and high (1000×) magnifications.

### 2.7. Isolation and Characterization of Bovine Myoblasts

A fresh bovine muscle (chunk roll) was obtained from River Bend Farm (Far Hills, NJ, USA). The isolation followed a previously reported procedure [39] with modifications (Supplemental Figure S1A). Briefly, the surface of the tissue was sterilized with 70% isopropanol. The surface tissues were removed in a biosafety cabinet and a few pieces of tissue were cut out from the center region and minced further into smaller pieces (<3 mm × 3 mm × 3 mm). The tissues were digested in collagenase digestion solution (DMEM base medium + 10% fetal bovine serum + 25 μg/mL gentamicin + 3 mg/mL collagenase type I (Worthington Biochemical Corporation, Lakewood, NJ, USA) at 3.5 mL/g of tissue. The digestion was incubated at 37 °C in a tissue culture incubator with rocking for 1 h. The digestion was diluted to 6x volume with PBS. The digestion was passed through 100 μm cell strainers and then 40 μm cell strainers. The passing was collected and the isolated cells were washed with PBS twice. Cells were either cryopreserved as P0 cells or plated onto gelatin-coated tissue culture-treated polystyrene (TCP) dishes in culture medium (DMEM + 20% FBS + 1x antibiotic/antimycotic (Thermo Fisher Scientific, Waltham, MA, USA). The cells were harvested from TCP dishes and labeled with PE anti-human CD56 (NCAM) Antibody (Clone MEM-188) (Biolegend, San Diego, CA, USA) and analyzed using a Beckman Coulter Gallios Flow Cytometer (Figure S1B). The CD56+ cells were plated at low density (50–100 cells/10 cm TCP dish) and cultured for 7–10 days. Colonies showing spontaneous fusions at a high cell density were isolated and cryopreserved (Figure S1C).

### 2.8. Culturing Bovine Myoblasts on Scaffolds

The collagen and ECM scaffolds underwent UV sterilization for 30 min on each side in a tissue culture hood. Bovine myoblasts were then seeded onto the scaffolds at a concentration of 10–15 × 10^4^ cells per scaffold in 100 µL of cell solution. Following seeding, the scaffolds were incubated at 37 °C for 15 min to facilitate cell attachment. Subsequently, the scaffolds were flipped using sterilized forceps, and another 100 µL of cell suspension at the same concentration was applied to the opposite side. After an additional 15 min incubation period for cell attachment, 0.5 mL of complete media was added to each well. The plates were then placed in a 37 °C tissue culture incubator with 5% CO_2_ and 95% humidity for further incubation.

### 2.9. Monitoring Growth of Bovine Myoblasts in Scaffolds

The growth of the cells seeded on the scaffolds was monitored using alamarBlue assay (Bio-Rad Laboratories, Philadelphia, PA, USA). alamarBlue assay was conducted at specific time points, where growth media were replaced with 0.3 mL/well of alamarBlue solution (complete growth medium + 10% alamarBlue reagent) and incubated at 37 °C for 30 min. After incubation, 0.1 mL of supernatant was transferred to a 96-well plate, and fluorescent intensity was measured using a multimode microplate reader (Spark^®^, TECAN, Switzerland) at excitation/emission wavelengths of 540 nm/590 nm, with fluorescent intensity reported in arbitrary units (AU).

To sustain cell proliferation on scaffolds, constructs were washed with 0.5 mL/well of PBS following the alamarBlue assay. Subsequently, samples were transferred into alpha-MEM complete media for an additional 1 h of incubation. After aspirating the complete media, 0.4 mL/well of fresh medium was added, and the cultures were continued for the next time point. alamarBlue assay was repeated to assess cell viability at specific time points, with results evaluated using the provided equation.

(1) $$Viability=\frac{{FL}_{\left(day\;T\right)}}{{FL}_{\left(day\;1\right)}}\;x\;100$$

### 2.10. Staining

#### 2.10.1. Immunofluorescent Staining

Immunofluorescent staining was performed, as previously described [40]. Briefly, scaffolds or cells were fixed with 4% paraformaldehyde for 1 h and permeabilized in 0.5% Triton X100 in PBS for 1 h. The fixed and permeabilized samples were stained with primary antibodies, which were diluted in staining buffer (1xPBS + 5% FBS + 0.02% NaN_3_), and the staining was incubated at 4 °C overnight. The primary antibodies used in this study are anti-bovine type I collagen polyclonal antibody (AB749P Millipore Sigma, Burlington, MA, USA) at 1:50 dilution and anti-bovine Desmin K5 polyclonal antibody (MUB0402S, Thermo Fisher Scientific, Waltham, MA, USA) at 1:100 dilution. After the incubation with primary antibodies, samples were washed and stained with secondary antibodies, goat anti-rabbit IgG-Alexa 555 antibody (Ref #A21428, Life Technology), or goat anti-mouse IgG-Alexa 488 (Ref#A21042, Life Technology, Carlsbad, CA, USA) at 1:500 for 1 h. Nuclei were stained with Hoechst dye 33258 in 20 mM of water (Cat #83219, AnaSpec Inc., Fremont, CA, USA) at 1:500 for 5–15 min. Staining samples were imaged under an epi-fluorescent microscope (Echo Revolve, San Diego, CA, USA).

#### 2.10.2. CalceinAM Staining

After a 30-day incubation of bovine myoblasts on ECM scaffolds, live staining was conducted using CalceinAM. Briefly, cells were treated with CalceinAM (1 μM in PBS, Corning Inc., Corning, NY, USA) for 30 min at room temperature, followed by PBS washes, and visualization using a fluorescence microscope (Echo Revolve, San Diego, CA, USA).

### 2.11. Design of Steak Construct

The steak-shaped mold for PDMS (positive mold) was designed using Autodesk Inventor Professional 2024 software (Autodesk, San Francisco, CA, USA) with specified dimensions (46 mm × 27 mm × 7 mm). The design was then exported to an STL file and imported into FormLabs Preform 3.34.1 software (Formlabs, Somerville, MA, USA) to configure printing settings and slice the model. Printing was performed with 100% density and a 0.100 mm layer thickness using Surgical Guide resin and the Form 3B SLA printer from FormLabs. Subsequently, the print underwent a 20 min wash in 99% isopropanol alcohol (VWR, Radnor, PA, USA) using Form Wash, followed by air drying and curing under UV light at 70 °C for 30 min using Form Cure. Finally, the supports were removed, and the model was utilized to prepare the PDMS mold.

### 2.12. Preparing PDMS Mold from 3D Printed Steak-Shaped Mold

Utilizing the Sylgard 184 Elastomer Kit (Electron Microscopy Sciences, Hatfield, PA, USA), PDMS was prepared by mixing silicone elastomer and curing agent in a 1:10 ratio until homogeneous, then placed in a vacuum oven for 30 min at RT under low vacuum to eliminate trapped air. The PDMS was carefully transferred into the 3D-printed steak-shaped scaffold mold and cured in a vacuum oven (Fisher Scientific, USA) at 80 °C for 2.5 h. Cured PDMS was peeled off from the mold and used in the preparation of steak-shaped scaffolds. 

### 2.13. Preparing BP-ECM for Steak-Shaped Scaffold

A 15 mg/mL BP-ECM solution was prepared by dissolving ECM in 10 mM HCl while mixing overnight at 4 °C. The fully dissolved ECM was then transferred into the PDMS mold, frozen at −20 °C, and lyophilized. The ECM scaffolds were removed from the PDMS mold and crosslinked in the vacuum oven at 140 °C for 24 h with the vacuum set to −20 Hg.

### 2.14. Pan Frying ECM Scaffolds Containing Bovine Myoblasts

The bovine ECM containing bovine myoblasts was cultured for 51 days with gentle shaking in the tissue culture incubator. The growth medium was changed every three days. To test if this meat construct can sustain the cooking process, BP-ECM containing bovine myoblasts was pan-fried with olive oil (Ward’s Science, Rochester, NY, USA) at 170 °C for 2 min on each side using a pan and IKA magnetic heater plate (Model C-MAG HS 7, IKA, Staufen, Germany).

### 2.15. Statistical Analysis

Each experiment included a minimum of three samples (n ≥ 3) as biological replicates, and findings are expressed as mean ± standard deviation. Statistical significance was assessed using one-way ANOVA with Tukey’s multiple comparisons test, using GraphPad Prism version 10.3.0 (461) (26 July 2024), GraphPad Software (La Jolla, CA, USA), with significance defined at *p* < 0.05.

## 3. Results

### 3.1. Isolation of Collagen and ECM from Bovine Placentome

**Figure 2:**
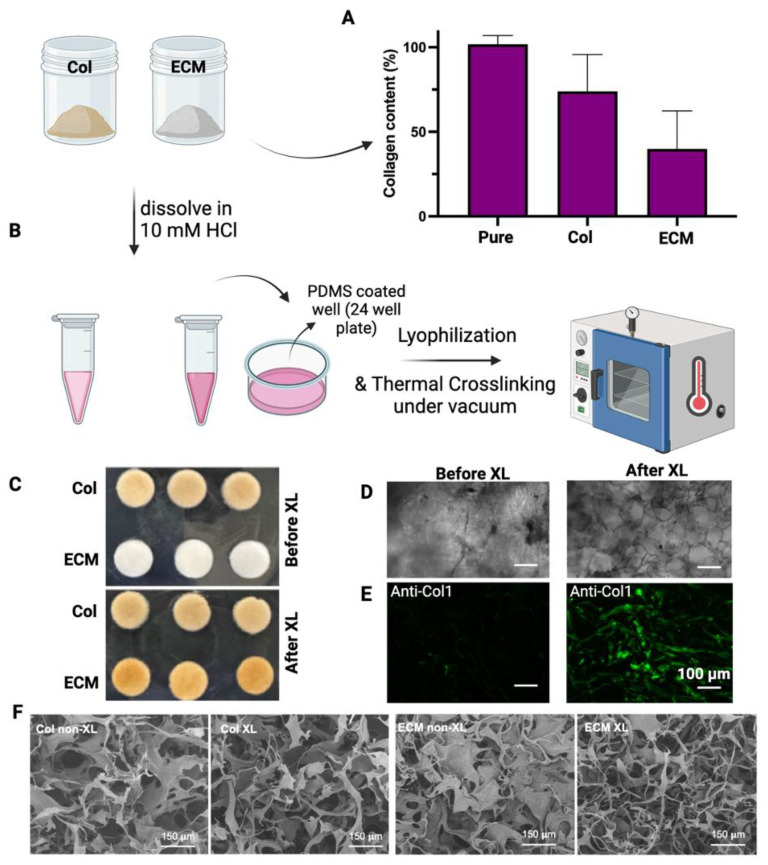
Characterization and fabrication of isolated collagens and ECM. (**A**) The collagen content of isolated collagen or ECM was determined by hydroxyproline assay. The collagen content (%) = weight of calculated collagen/dry weight of sample. Data are shown as mean ± SD (n = 4). (**B**) A schematic representation of the crosslinking process is presented. (**C**) Images display collagen and ECM scaffolds both before and after crosslinking. (**D**) Bright-field images showcase the morphologies of ECM scaffolds before and after crosslinking. (**E**) Fluorescence staining against bovine collagen type I shows the fibrillar structure of the scaffolds after crosslinking. Scale bar = 100 µm. (**F**) Representative SEM images of non-crosslinked (non-XL) and DHT crosslinked (XL) scaffolds. Scale bar = 150 µm.

Collagens were isolated from bovine placentomes, as described in Methods. During the collagen isolation process, a series of sequential filtration steps were performed before salt precipitation. The digested solutions were filtered through filters with pore sizes of 20 µm, 10 µm, and 5 µm. After filtering through a 5 µm filter, the solution looked clear with a slight brownish color. Unlike the isolation of collagen from the bovine placentome, the total ECM was collected from the digestion of decellularized bovine placental tissues. The collagen contents in collagen preparations and ECM were assessed using hydroxyproline assay (Figure 2A). The purity of the isolated collagen was around 75%. The collagen content in ECM was about 40%, suggesting that there are other ECM components in this preparation. To evaluate if these different collagen/ECM preparations have differential effects on the fabrication and bioactivity of scaffolds, these samples were subsequently employed to fabricate porous scaffolds for making cultivated meat. After dissolving collagen/ECM in 10 mM HCl at 4 °C, freezing in PDMS-coated well plates, and crosslinking at 150 °C under vacuum for 24 h (Figure 2B), collagen scaffolds maintained their color, while ECM scaffolds transitioned from white to brownish color (Figure 2C). Bright-field microscopy (Figure 2D) and fluorescence staining against bovine collagen type I (Figure 2E) revealed the formation of pore structures and fibrillar networks in scaffolds upon DHT crosslinking. Consistent with this observation, scanning electron microscopy (SEM) analysis of the scaffolds before and after crosslinking revealed an increase in fibrillar structures within the collagen or ECM scaffolds post-crosslinking (Figure 2F). The transition from a more sheet-like to a fibrillar structure, along with the enhanced antibody detection, may be attributed to DHT treatment. This treatment likely caused collagen fibers to become more tightly packed and aligned, increasing their density within the scaffold and enhancing the detection and signal of collagen type I. Knitlova et al. utilized second harmonic generation (SHG) imaging to detect collagen type I and observed a significantly reduced SHG signal when cells were treated with the LOX inhibitor β-aminopropionitrile (BAPN) to reduce collagen crosslinking in vitro, highlighting the critical role of crosslinking in the detection of collagen type I [41]. 

### 3.2. Myoblast Culture on Collagen and ECM Scaffolds

**Figure 3:**
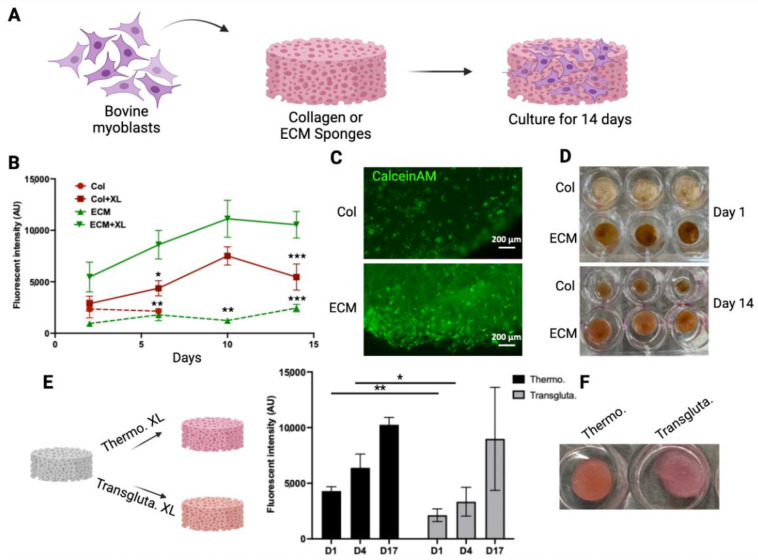
Bovine myoblast culture on collagen and ECM scaffolds. (**A**) A schematic representation of the bovine myoblast culture on the scaffolds. (**B**) The viability of bovine myoblasts cultured in crosslinked (XL) or non-crosslinked (NXL) scaffolds were monitored using alamarBlue assay for 14 days. The viability was expressed as the fluorescent intensity (arbitrary units). Data shown are mean ± SD (n = 3). * *p* < 0.05, ** *p* < 0.0, *** *p* < 0.005 compared with the viability of ECM + XL. (**C**) The viability of cells in crosslinked collagen scaffold (upper) and ECM scaffold (lower) was detected by CalceinAM staining. (**D**) The shapes of the scaffolds over time. The crosslinked scaffolds with cells were cultured for 1 day and 14 days. (**E**) ECM scaffolds were crosslinked using thermal crosslinking (Thermo. XL) or transglutaminase (Transgluta. XL). Viability of cells in crosslinked ECM scaffolds was monitored over time. Data shown are mean ± SD (n = 3) * *p* < 0.05, ** *p* < 0.01. (**F**) Images of scaffolds containing cells on Day 17. Scale bar = 200 µm.

To evaluate if these scaffolds have the potential to serve as scaffolds for making muscle tissue for cultivated meat, the growth of bovine myoblasts in these scaffolds was monitored and compared (Figure 3A). To demonstrate the impact of crosslinking on cell growth, both crosslinked and non-crosslinked scaffolds were tested. The viability of cells in the scaffolds was monitored using an alamarBlue assay for 14 days (Figure 3B). The crosslinked collagen scaffolds sustained the 14-day culturing period, whereas the non-crosslinked collagen scaffolds disintegrated after 3 days. Cells showed significantly higher viability in crosslinked ECM scaffolds compared with cells on other scaffolds. The crosslinking of ECM significantly enhanced the biocompatibility of scaffolds with cells (Figure 3B). The viability of cells in crosslinked scaffolds was also assessed by CalceinAM staining (Figure 3C). The intensity and density of viable cells (green) were higher in crosslinked ECM scaffolds. The growth of cells in the scaffolds resulted in the contraction of collagen scaffolds (compare the upper and lower panels of Figure 3D). On the other hand, the structures of ECM crosslinked scaffolds were maintained over the culturing period with only a slight reduction in size (from 12 mm to 10 mm). These results suggested that the crosslinked ECM may serve as a scaffold for culturing bovine myoblasts. Other than DHT crosslinking, transglutaminase crosslinking has been used widely as a biocompatible crosslinking agent for biological molecules and materials [42]. To compare these two crosslinking methods, ECM scaffolds were crosslinked using either thermal crosslinking or transglutaminase crosslinking. The growth of bovine myoblasts within the crosslinked scaffolds was then compared (Figure 3E). Cell viability in thermal crosslinked ECM scaffolds was consistently higher than that in transglutaminase crosslinked scaffolds at all time points. Additionally, the transglutaminase crosslinked scaffolds exhibited a softer and looser network structure compared to the thermal crosslinked scaffolds (right panel, Figure 3E). These results indicate that DHT crosslinking not only produces stable and biocompatible ECM scaffolds but also eliminates the need for additional chemicals or enzymes. Therefore, the DHT crosslinking method was chosen for crosslinking bovine ECM scaffolds in further studies.

### 3.3. Making a Muscle Tissue Like Meat Construct

**Figure 4:**
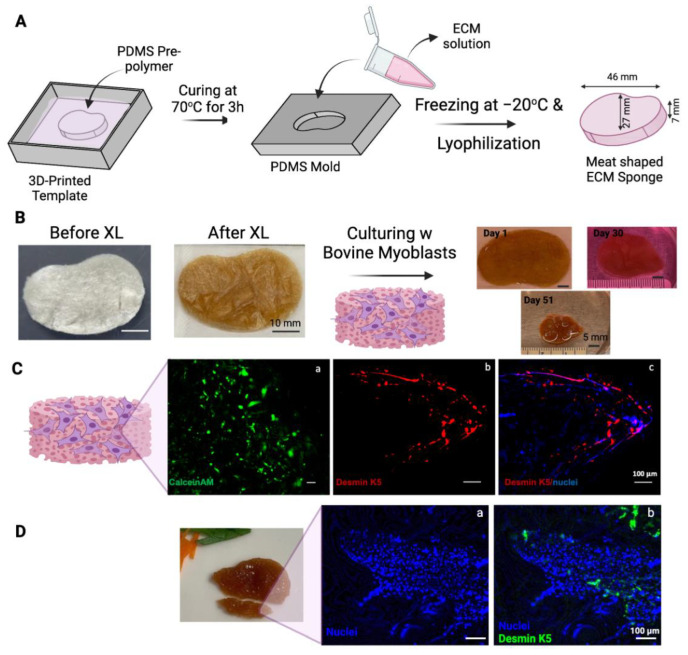
Steak-shaped 3D-printed mold and cell culture in the scaffold. (**A**) Schematic representation of the fabrication process for the steak-shaped scaffold. (**B**) Images depict steak-shaped ECM scaffolds both before and after crosslinking (scale bar is 10 mm), along with their appearance during cell culture for 51 days (scale bar is 5 mm). (**C**) Calcein AM (green, (a)), Desmin (red, (b)), and Nuclei (blue, (c)) staining on Day 30. (**D**) Desmin (green, (a)) and Nuclei (blue, (b)) staining on Day 51. The scale bar is 100 μm.

In an attempt to make a muscle-like tissue to mimic a miniature steak, a steak-like shape scaffold made of ECM was prepared using Autodesk Inventor 2023 (Autodesk, San Rafael, CA, USA) (Figure 4A). A FormLabs SLA printer was employed to 3D-print a mold with the dimensions specified in Figure 4A. A negative mold was obtained using PDMS, and ECM scaffolds were produced accordingly. The 1.5 × 10^6^ bovine myoblasts (P3) were seeded onto the thermal crosslinked ECM scaffold (scaffold dry weight = 75 mg). The scaffold-containing cells were cultured for a total of 51 days with gentle shaking in the tissue culture incubator (Figure 4B). The overall size of the construct shrank over the 51 days of culturing (some samplings were taken over the incubation time to determine the cell viability). Interestingly, without any artificial coloring, the color of the construct seemed similar to that of the muscle tissue of steak meat. Currently, whether the expression of heme, darkening of the polysaccharides in the crosslinked ECM scaffold, or other factors contributing to this meaty color is under investigation.

The viability of myoblasts in scaffolds was evaluated by staining a sample from the construct on Day 30 with CalceinAM (Figure 4C(a)). Viable cells were detected throughout the scaffold. The sample was also fixed and immunostained with antibodies against bovine Desmin k5, a marker of myogenic differentiation. As shown in Figure 4C(b,c), the expression of desmin k5 was detected in the scaffold, especially along the edges of the scaffold. This preferential myogenic differentiation of myoblasts along the edge of the construct may be due to the shear stress generated along the edge of the construct by the shaking during the culturing.

The cellularity in the scaffold increased from Day 30 (Figure 4C(c)) to Day 51 (Figure 4D), as demonstrated by the staining of the nuclei with Hoechst dye. The increased cell density may partially be attributed to the condensation of the construct and the growth of cells over time.

**Figure 5:**
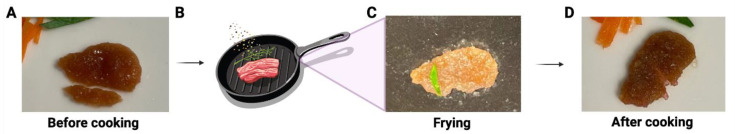
Illustration of the proof-of-concept of frying lab-cultivated meat with cooking oil. (**A**) The cultivated meat before cooking (one small piece below was cut and removed from the whole piece for other testing). (**B**) Schematic illustration of frying. The representative image of the cultivated meat during (**C**) and after (**D**) frying.

This construct not only showed a similar color to the muscle tissue of real meat, but it also had a texture similar to that of a thin slice of real meat during handling (Supplemental Video S1). Furthermore, this meat construct has also been tested for grilling/frying. As shown in Figure 5 and Supplemental Video S2, the meat construct maintained its shape after frying in olive oil for 2 min. The sizzling and smell during the cooking was like the cooking of real meat.

## 4. Discussion

In vitro cultivated meat, an emerging technology, involves producing edible muscle tissue in a laboratory setting and utilizing biotechnological tools for synthetic tissue production. Cultivated meat offers benefits such as improved animal welfare, enhanced human health by mitigating risks like transmissible spongiform encephalopathy and foodborne hazards such as salmonellosis, and contributes to environmental sustainability by reducing impacts like global warming [43,44,45]. The core objectives of this technology include culturing muscle progenitor cells without animal slaughter, designing edible scaffolds conducive to myoblast proliferation, formulating serum-free cell culture media, and utilizing bioreactors for myogenic stimulus application to obtain muscle fibers [21]. This study aims to contribute to the second objective by developing an edible 3D porous construct, where anchorage-dependent cells such as muscle cells can remain viable and proliferate. Selecting an optimal scaffolding material is crucial for cultivated meat production. Animal-derived materials, while effective at mimicking the extracellular matrix, face environmental and ethical issues. Plant-derived scaffolds offer tunable properties essential for cell penetration and nutrient supply but may lack specific cell adhesion motifs and vary in mechanical properties. Synthetic polymers, although customizable, might not fully replicate the natural cellular environment and can affect the taste of the final product [46]. The main challenge is ensuring scaffold size and thickness support cell viability, requiring innovations in vascularization or perfusion channels to overcome oxygen and nutrient diffusion limits [30]. Among all the studied scaffolding materials, collagen, the primary constituent of the extracellular matrix (ECM), finds widespread use across various industries including food, medicine, cosmetics, cell culture, leather, film, pharmaceuticals, and biomedical materials [47,48]. While most commercial collagen products are sourced from slaughtered pigs and cows [48], which perpetuates animal dependence, our utilization of collagen-rich bovine placentomes addresses both environmental and ethical concerns. These placentomes naturally form and are expelled postpartum without causing harm to the livestock [49,50], aligning with the objective of in vitro meat production to eliminate the need for mammal slaughter.

The collagen utilized in this study was isolated following a previously established protocol. Initially, the tissues underwent lyophilization and grinding to enhance protein extraction efficiency. The extraction process commenced with osmotic shock induced by concentrated NaCl solution, followed by sterilization with 0.5 N HCl. Subsequent pepsin digestion resulted in a brownish slurry, indicating residual blood particles, which were eliminated through sequential filtration. The final filtration, with a pore size of 5 µm, yielded a slightly brownish-colored solution. The collagen was then precipitated using concentrated NaCl, followed by 3–4 days of dialysis at 4 °C against 10 mM HCl to remove salt residues from the precipitate.

The process of ECM extraction began with SDS treatment to decellularize the tissue, followed by tissue grinding post-lyophilization to optimize extraction. Detailed investigations by McCabe et al. demonstrated a notable enhancement in ECM coverage through both decellularization and chemical digestion [51], as utilized in this study. Subsequent pepsin treatment was followed by neutralization to pH 7, and dialysis against diH_2_O at 4 °C to eliminate any salts entrapped in the slurry.

The quantification of collagen in the ‘collagen’ preparation used in this study revealed that approximately 75% of the dry mass was pure collagen. The identities of the remaining 25% of non-collagen components were unclear. It is likely that these non-collagen fractions consisted of partially digested tissue particles (smaller than 5 μm). By removing particles using a 0.22 μm filter before precipitation, the total collagen content could be increased to nearly 100% pure collagen (Figure 2A). However, crosslinked scaffolds made of pure collagen showed significant contraction in the presence of bovine myoblasts within a few days, similar to previous reports [52,53]. In comparison, scaffolds made of non-pure collagen materials (filtered at 5 μm) demonstrated better stability during long-term culturing. Therefore, non-pure collagen materials were used for this study. The ECM preparation isolated from bovine placentomes contained approximately 40% pure collagen. The other 60% non-collagen components likely included various ECM proteins, polysaccharides, and undigested tissue particles. Although their exact identities are unclear, these components played positive roles in maintaining the stability, biocompatibility, and coloring of the scaffolds.

The extracted collagen and ECM were employed in scaffold fabrication for cultivated meat production. An ideal scaffold should possess qualities such as biocompatibility, biodegradability, affordability, porosity, texture, and non-toxicity [30]. In this regard, scaffolding technologies originally developed for tissue engineering can be adapted to cultured meat production by utilizing edible materials and avoiding toxic crosslinkers or solvents [54]. Various technologies, including microcarriers, nanofibrous scaffolds, hydrogels, decellularized scaffolds, porous scaffolds, and scaffold-free methods, are being explored [46]. For example, Yen et al. recently developed a platform that integrates edible microcarriers and an oleogel-based fat substitute, expanding bovine MSCs on chitosan-collagen microcarriers to create cellularized microtissue [55]. Hydrogels, with their high water content, tunable mechanical properties, and biocompatibility, provide an environment conducive to cell growth and differentiation, as demonstrated by Rao et al. with their gelatin-based hydrogels using grape seed extract [56]. Despite their potential, most scaffolds face challenges in achieving suitable mechanical properties, biocompatibility, scalability, cost-effectiveness, and long-term stability [46]. Sponges generated through the freeze-drying technique emerge as top candidates for porous scaffolds in tissue engineering applications due to their simplicity, lack of dependency on organic solvents, and ease of molding into various shapes [57]. This technique is well-established for crafting collagen-based biomaterial scaffolds featuring a porous structure while preserving the bioactive properties of collagen [58]. In our study, we prepared PDMS-coated 12-well plates to serve as molds for the collagen and ECM sponges. PDMS facilitated easy removal of the scaffolds from the surface without causing damage. To improve scaffold stability throughout the culturing period, we implemented DHT(DHT) treatment using previously optimized parameters [36]. DHT, a widely employed method for crosslinking type I collagen, involves the removal of water from collagen at high temperatures under low pressure, leading to intermolecular crosslinking through condensation reactions, namely amide formation or esterification [59,60]. This approach distinguishes itself as a safe technique for food processing, as it circumvents the need for toxic crosslinkers, boasts gentler action compared to chemical alternatives, is cost-effective, and extends scaffold biodegradation [59,61]. Additionally, DHT treatment was reported to improve the structure and mechanical properties of the scaffolds [38,61].

The application of DHT treatment had no impact on the color of the collagen scaffolds, whereas the color of ECM scaffolds transitioned from white to brownish, indicating the presence of additional ECM components, such as glycosaminoglycans, influenced by the DHT treatment. Štiglic et al. (2021) observed a gradual color change from yellow to brown in polysaccharide-based scaffolds composed of carboxymethylcellulose and chitosan, particularly pronounced at 120 °C [62], attributed to the formation of degradation products [62,63]. Although partial denaturation resulting from thermal crosslinking can render scaffolds more susceptible to enzymatic degradation, which is potentially undesirable for tissue engineering, it may offer advantages for food applications, enhancing digestibility with enzymes such as trypsin [64] and pepsin [65]. The brownish color of the ECM scaffolds post-crosslinking also conferred a meat-like appearance, eliminating the need for artificial coloring.

DHT crosslinking significantly improved the stability of collagen scaffolds, as evidenced by the rapid dissolution/degradation of uncrosslinked counterparts within a week of myoblast incubation. Cell proliferation was positively influenced by crosslinking in both scaffold types, with ECM scaffolds demonstrating superior support for cell growth compared to collagen scaffolds (Figure 3C). Furthermore, while collagen scaffolds exhibited shrinkage over the 14-day incubation period (Figure 3D), ECM scaffolds maintained their stability with a slight reduction in size. Shrinkage represents a critical drawback of collagen sponges, impacting pore, oxygen, and nutrient availability for cells [66,67] which likely accounts for the observed lower cell proliferation on collagen scaffolds compared to ECM scaffolds. Furthermore, compared to an enzyme-mediated crosslinking method, DHT crosslinked scaffolds exhibited superior biocompatibility, stability, and texture compared to transglutaminase crosslinked scaffolds. Consequently, DHT crosslinked bovine ECM scaffolds were selected for creating meat constructs in this study.

One advantage of the freeze-drying technique is its ability to dictate the final geometry of the biomaterial solution based on the shape of the container or mold used for fabrication [58]. In this study, we designed a steak-shaped model as detailed in Section 3.3 The model was then 3D printed using Formlabs with a biocompatible surgical guide resin, and the resulting print was used to create a PDMS-positive mold for producing steak-shaped sponges. The ECM scaffolds obtained from this mold were subsequently crosslinked with DHT treatment, resulting in a brownish color attributed to partial degradation of glycosaminoglycans within the ECM structure, imparting a meat-like appearance after culturing without additional food coloring. Over the 51-day culturing period, the overall scaffold size decreased, likely due to cell-induced contraction. CalceinAM and nuclei staining on Day 30 and nuclei staining on Day 51 revealed increased cell abundance within the structure, with positive desmin k5 staining on both days indicating myogenic differentiation of the myoblasts. The majority of positive staining was observed along the construct’s edges, suggesting the influence of shear stress generated during culturing under agitation. Further enhancement of cell penetration depth, cell number, and differentiation can be achieved through bioreactor utilization and optimization of culture conditions and implanting mechanical stimulations [68]. While agitation culturing, as employed in this study, facilitates homogeneous media access and generates shear stress around the scaffolds, it lacks precise control over nutrient, oxygen, and waste transport, rendering it suitable only for laboratory-scale applications [12]. Sinlapabodin et al. (2015) utilized perfusion bioreactors in their study to investigate the osteogenic differentiation of rat bone marrow-derived stem cells on silk fibroin/gelatin/hydroxyapatite scaffolds [69]. They found that a moderate flow rate of 3 mL/min resulted in the highest osteogenic differentiation and significantly increased ALP enzyme activity and calcium content compared to dynamic conditions. The design of bioreactors is a critical aspect of scaling up cultivated meat production and commercializing technology. Computational fluid dynamics models, commonly used for simulating bioreactors in various applications, will play a vital role in scaling up the technology by determining optimal processing parameters for cultivated meat production [70].

The texture of the construct we developed closely resembles real meat after 51 days of culturing, exhibiting robustness when handled and fried in olive oil for 2 min (Figure 5), emitting the same sizzling sound and aroma as real meat. Although we have demonstrated proof-of-concept meat constructs, more extensive studies are necessary to physically and mechanically characterize and optimize ECM scaffolds, as well as quantitatively assess texture and flavor in a scaled-up setting.

Despite the promise of cultivated meat, the industry remains at the early stage, with numerous challenges to overcome, including metabolic inefficiency, shear-induced cell damage, low growth rates, and high costs that hinder the scalability of bioreactors [12]. The field of cultivated meat represents both excitement and ambition, necessitating collective efforts from the scientific community to realize the vision of slaughter-free food products.

## 5. Conclusions

The emergence of in vitro cultivated meat technology holds significant promise in addressing various challenges facing traditional meat production systems. By producing edible muscle tissue in a controlled laboratory setting, this technology offers compelling advantages, including improved animal welfare, mitigation of environmental impacts, and reduction in foodborne hazards. Our study specifically contributes to advancing the field by focusing on the development and evaluation of edible scaffolds, a crucial component in the cultivation of muscle tissue.

Through meticulous extraction and fabrication processes, we have demonstrated the viability of using collagen-rich bovine placentomes as a sustainable and ethical biomaterial source for scaffold production. The successful utilization of these scaffolds in cultivated meat production represents a significant step forward in the quest for sustainable and slaughter-free meat alternatives.

However, as with any emerging technology, challenges persist. Issues such as scalability and costs remain hurdles that must be addressed to fully realize the potential of cultivated meat on a global scale. Continued research and collaboration within the scientific community are essential to overcome these challenges and advance the cultivated meat industry.

In conclusion, while the cultivated meat field is still in its early stages, the progress made thus far is promising. With further innovation, optimization, and collaboration, we can work towards a future where slaughter-free food products are not only achievable but also sustainable and widely accessible.

## Data Availability

Raw data are included in Supplemental Materials.

## Supplementary Materials

The following supporting information can be downloaded at https://www.mdpi.com/article/10.3390/bioengineering11080854/s1, Figure S1: Isolation and characterization of bovine myoblasts; Raw data files. Video S1/S2: The Z-stack of CalceinAM stained cells/ECM scaffold construct.
