# Supplementary material for: Bovine Placentome-Derived Extracellular Matrix: A Sustainable 3D Scaffold for Cultivated Meat
Source: Bioengineering (Basel). 2024 Aug 21;11(8):854. doi: 10.3390/bioengineering11080854 (PMC11352162; doi:10.3390/bioengineering11080854)
Supplement: Supplementary file 1 [file bioengineering-11-00854-s001.zip › bioengineering-3140463-supplementary-Figure S1.pdf]

Supplemental Figure S1

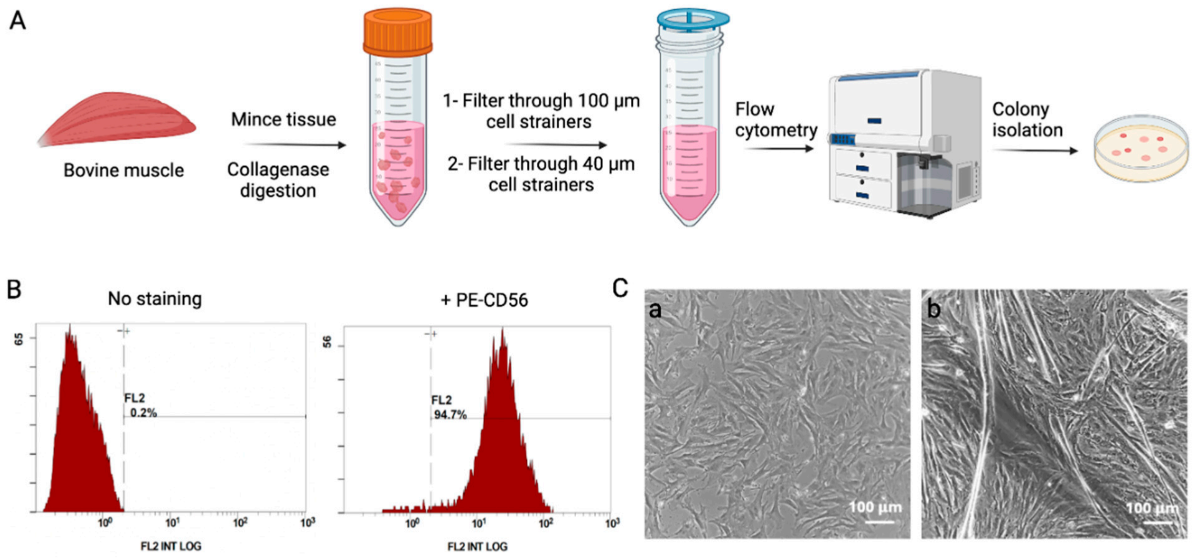

Figure S1. Isolation and characterization of bovine myoblasts. A fresh bovine muscle (chunk roll) was obtained from River Bend Farm (Far Hills, NJ). The isolation followed a previously reported procedure (Spinazzola JM and Gussoni E. Vol.7, Iss 21,2017. www.bio-protocol.org/e2591) with modifications (A). Briefly, the surface of tissue was sterilized by 70% isopropanol. Removed the surface tissues in a biosafety cabinet and cut out a few pieces of tissue from the center region and minced them further into smaller pieces (<3mmx3mm x3mm). Digested tissues in collagenase digestion solution (DMEM base medium+10% fetal bovine serum + 25µg/mL gentamicin + 3 mg/mL collagenase type I (Worthington Biochemical Corporation, Lakewood, NJ) at 3.5 mL/ gram of tissue. Incubated the digestion at 37°C tissue culture incubator with rocking for 1 h. Diluted the digestion 6x volume with PBS. Passed the digestion through 100 µm cell strainers then 40 µm cell strainers. Collected the passing through and washed the isolated cells with PBS twice. Cells were either cryopreserved as P0 cells or plated onto gelatin coated tissue culture treated polystyrene (TCP) dishes in culture medium (DMEM+20% FBS+1x antibiotic/antimycotic (Thermo Fisher Scientific, Waltham, MA). Harvested cells from TCP dishes and labeled with PE anti-human CD56 (NCAM) Antibody (Clone MEM-188) (Biolegend, San Diego, CA) and analyzed using Beckman Coulter Gallios Flow Cytometer (B). The CD56+ cells were plated at low density (50-100 cells/10 cm TCP dish) and cultured for 7-10 days. Colonies showed spontaneous fusions at high cell density were isolated and cryopreserved (C).
